# Streptococcus Pneumoniae Causing Intra-abdominal and Pelvic Infection: A Case Series

**DOI:** 10.7759/cureus.1967

**Published:** 2017-12-19

**Authors:** Amrita Ronnachit, Katherine A Ellenberger, Timothy J Gray, Eunice Y Liu, Timothy L Gilbey, Elaine Y Cheong, Thomas Gottlieb, Genevieve L McKew

**Affiliations:** 1 Department of Microbiology and Infectious Diseases, Concord Repatriation General Hospital; 2 Department of Microbiology and Infectious Diseases, Westmead Hospital

**Keywords:** abdomen, pelvis, infection, streptococcus pneumoniae

## Abstract

Streptococcus pneumoniae is a pathogen known to cause pneumonia, sinusitis, meningitis, and otitis media, but is overlooked as a pathogen causing gastrointestinal illness. We report four cases of Streptococcus pneumoniae causing intra-abdominal and pelvic infection. Streptococcus pneumoniae should be considered in the setting of intra-abdominal infection, especially in patients with risk factors for invasive pneumococcal disease or with a concomitant respiratory infection at presentation.

## Introduction

Streptococcus pneumoniae (S. pneumoniae) is a pathogen known to cause pneumonia, sinusitis, meningitis and otitis media [1]. Pneumococcal infection can also be associated with nonspecific abdominal symptoms, such as nausea, vomiting and diarrhoea [2]. It is, however, an under-recognized cause of primary intra-abdominal and pelvic infections. It has been reported to cause primary peritonitis, salpingitis, enteritis and appendicitis in adults and children [2-4]. We present here four cases of intra-abdominal and pelvic infections associated with S. pneumoniae bacteremia.

## Case presentation

Case one

A 37-year-old female presented with fever, vomiting, diarrhoea, generalised myalgias and coryza. She was discharged with a suspected viral illness but was readmitted when blood culture isolated S. pneumoniae. She had right lower quadrant pain, was tachycardic and hypotensive. An examination demonstrated generalised abdominal tenderness, with signs of peritonism in the right iliac fossa. Her C-reactive protein (CRP) and white cell count (WCC) were elevated (Table 1). At emergency laparoscopy and appendicectomy, there was purulent exudate throughout the abdomen, with an unremarkable appendix and inflammation of the right fallopian tube. A urine pneumococcal antigen was positive (Alere BinaxNOW (Alere Inc., Massachusetts, United States) immunochromatographic antigen assay was used in all four cases). Neisseria gonorrhoeae and Chlamydia trachomatis were not detected in urine by PCR testing. The patient was treated with intravenous benzylpenicillin and discharged well on day six.

**Table 1:** Patient summary and investigations ^a^Systemic Lupus Erythematosus, ^b^Large Loop Excision of the Transformation Zone, ^c^Polymerase Chain Reaction, ^d^Antigen, ^e^Red Blood Cell, ^f^ White Blood Cell

	Case 1	Case 2	Case 3	Case 4
Age (years) Gender (M/F)	37 F	40 F	41 F	49 F
Presenting symptoms	Vomiting, diarrhoea, fever, abdominal pain, coryza, myalgia,	Vomiting, diarrhoea, fever, abdominal pain	Vomiting, diarrhoea, fever, abdominal pain	Diarrhoea, fever, abdominal pain, myalgia, headache
Medical and surgical history	SLE^a^, pericarditis, pleuritis, depression, LLETZ^b^ procedure for cervical intraepithelial neoplasia 10 years prior.	Splenectomy for idiopathic thrombocytopaenic purpura 10 years prior, ruptured ectopic pregnancy, cholecystectomy and appendectomy. Penicillin allergy	Proctitis. Penicillin allergy	Nil significant
Peak CRP (mg/L)	316	153	>250	485
Lactate (mmoL/L)	1.2	4.6	Not done	3.3
Peak/ WCC (10^9^/L)	17.5	43	30	17.5
Pneumococcal vaccination status	Unknown	Pneumovax23 (Merck) 3 years prior	Unknown	Not vaccinated
Intrauterine Device present (yes/no)	No	No	Yes	No
Regular Medications	Prednisone 5 mg Escitalopram	Prednisone 25 mg	Mesalazine	Nil
Clinical disease	Peritonitis, Salpingitis	Sigmoid colitis, pneumonia	Enteritis, peritonitis	Enteritis
Bacteraemia	S. pneumoniae 15B	S. pneumoniae 23B	*S.* *pneumoniae* 3	*S.* *pneumoniae* 3
Positive microbiology for pneumococcus	Pelvic pus PCR^c^,Urine Ag^d^, Ascites Ag		Urine Ag, Blood Ag, Stool Ag, Ascites Ag	Urinary Ag, Stool Ag
Stool investigations			Predominant gram-positive diplococci on stool microscopy	Stool microscopy and culture negative with nil organisms, RBC^e^ or WBC^f^ seen
Directed treatment	Exploratory laparotomy. Benzyl penicillin	Moxifloxacin	Cefotaxime and lincomycin. Drainage of intra-abdominal collections	Ceftriaxone and metronidazole

Case two

A 40-year-old female presented with acute onset central abdominal pain with vomiting and diarrhoea. She was febrile (39.0°C) and tachycardic (120 bpm). On examination, there was a reducible hernia in the right iliac fossa and marked tenderness in the epigastrium and right upper quadrant without peritonism. Her inflammatory markers were elevated (Table 1). A computed tomography scan (CT) of the abdomen and pelvis demonstrated mural thickening of the sigmoid colon, and a chest X-ray demonstrated left perihilar opacity. She was treated for sigmoid colitis with ceftriaxone and metronidazole. She developed hypotension, which was treated with intravenous fluids. On the fourth day of admission, blood cultures collected at presentation isolated S. pneumoniae. Treatment was changed to moxifloxacin once daily, and she was discharged. Subsequent colonoscopy and gastroscopy demonstrated no evidence of colitis or colonic abnormalities.

Case three

A 41-year-old woman presented with a four-day history of abdominal pain, vomiting and diarrhoea, with fever and rigors for 24 hours. She appeared unwell with a heart rate of 115 bpm and BP of 108/70. She had diffuse abdominal tenderness without any clinical evidence of peritonitis. Her inflammatory markers were elevated (Table 1). Her previous medical history included undifferentiated proctitis managed with mesalazine and an intrauterine contraceptive device (IUD) inserted three months prior to her presentation. She was commenced on intravenous ciprofloxacin for a presumptive diagnosis of infective colitis. She developed hypotension requiring fluid resuscitation. A CT scan revealed thickened loops of the small bowel with peritoneal enhancement and bilateral pleural effusions. Blood cultures isolated S. pneumoniae serotype 3. She initially improved when commenced on cefotaxime and lincomycin. However, on day seven of admission, her dyspnoea worsened and she developed diffuse erythema on the anterior abdominal wall. Thoracocentesis revealed a sterile transudate, however, an abdominal paracentesis performed on day 11 of admission revealed purulent fluid (leucocytes 54 x 10^9/mL) with glucose of 0.1 mmol/L and a low serum-ascites albumin gradient (6 g/L). Predominant gram-positive diplococci were noted on stool microscopy. Urine, stool, pleural and ascitic fluid were positive on pneumococcal antigen testing. Despite 15 days of treatment with cefotaxime, her abdominal symptoms persisted and she underwent laparotomy to drain sterile intra-abdominal abscesses. She was discharged well on day 30.

Case four

A 49-year old woman presented with a four-day history of profuse, watery diarrhoea, abdominal pain, fevers and rigors. She appeared unwell, with a respiratory rate of 22, heart rate (HR) of 90 and signs of dehydration. Widespread abdominal tenderness was present without peritonism, and some bloating was noted. See Table 1 for investigations. She was commenced on empiric ciprofloxacin and metronidazole. Blood cultures isolated S. pneumoniae and the pneumococcal antigen was positive in urine and, although a non-validated specimen type, was positive in stool. Antibiotics were changed to ceftriaxone and metronidazole. On day four of her admission, she developed melaena. A CT of the abdomen demonstrated enteritis with a thickened bowel wall, widespread ascites and bilateral pleural effusions. She improved and was discharged well on day nine 9.

## Discussion

We describe a series of four women with pneumococcal abdominopelvic infections with a spectrum of disease ranging from enteritis to severe purulent peritonitis. All four patients presented with abdominal complaints and were treated empirically for infectious colitis. One patient had peritonism and was treated for perforated abdominal viscus. Two of the patients (cases one and two) were prescribed long-term prednisone, and one patient (case two) was previously surgically splenectomised. All the cases had confirmed bacteraemia with S. pneumoniae, with serotypes 15B, 23B and 3, and all four cases were treated with antibiotics directed at S. pneumoniae once a microbiological diagnosis was made. One patient required exploratory and therapeutic laparoscopy and washout. All four patients recovered, although one patient required re-admission for laparotomy and drainage of abdominal collections.

Primary pneumococcal peritonitis has been described in children, and more rarely in adults [5]. Risk factors include nephrotic syndrome or cirrhosis and, to a lesser extent, peritoneal dialysis, rheumatoid arthritis or bone marrow transplant [5-6]. There have been case reports of primary pneumococcal peritonitis in patients with systemic lupus erythematosus (SLE). This is relevant to case one, where it is not clear from intraoperative findings whether she had primary pneumococcal peritonitis or salpingitis with secondary peritonitis. In this patient group, an important differential is lupus peritonitis, usually treated with systemic steroids and immunosuppressants, which are contraindicated in cases of bacterial peritonitis [7].

The mode of entry of S. pneumoniae into the peritoneal cavity is contentious, with possible routes including hematogenous spread, transmural intestinal translocation and translocation from the genital tract [4,8].

No patient in this series reported respiratory symptoms, but one (case two) had an infiltrate on chest x-ray, raising the possibility of hematogenous spread from a respiratory focus, with secondary enteritis. In this case, there was clear evidence of concomitant gastrointestinal abnormalities on CT in addition to a pulmonary infiltrate, distinguishing it from non-specific gastrointestinal symptoms that may accompany pneumococcal pneumonia [2,8].

Transmural translocation from the gastrointestinal tract is also postulated. Cleveland et al. reported that 24% of patients with bacteraemic pneumococcal pneumonia had diarrhoea [9], theorising that the pathogenesis may be related to a secretory diarrhoea without direct invasion of S. pneumoniae into the bowel wall. This is distinguished from the disease caused by a direct pneumococcal invasion of the intestine, which may present with appendicitis, enteritis or peritonitis. S. pneumoniae has been reported as a rare cause of acute appendicitis and could thereby cause secondary peritonitis [2,5]. However, it is not clear to what extent S. pneumoniae may colonise the gut and through what mechanisms it may invade the bowel mucosa [2].

In adults, there is a female preponderance of cases, as highlighted by the case series presented here [4-5]. The female preponderance of pelvic and abdominal pneumococcal infection raises the possibility that translocation from the female genital tract may be an important route of invasive disease [4-5]. An ascending infection may lead to pelvic inflammatory disease, including endometritis or salpingitis, and thereby to peritonitis [1]. S. pneumoniae is inhibited by an acidic environment [10] and is not part of resident genital tract flora. However, there may be transient colonisation of the vagina [3], perhaps facilitated by orogenital sexual practices, transmission from the respiratory tract via hand to genital contact or hematogenous spread [3]. Of note, case three presented with an intrauterine device (IUD), which has previously been suggested as a risk factor that may promote an ascending pneumococcal infection, especially in women with retrograde menstruation [3-4]. Other potential risk factors include recent gynaecological surgery or vaginal delivery, neither of which is relevant in our case series [4].

In Australia, the introduction of the 7-valent pneumococcal conjugate vaccine (7xPCV) has led to a significant reduction in the rates of invasive pneumococcal disease (IPD). Two cases presented here were at increased risk for IPD due to underlying immunocompromise secondary to prednisone therapy (cases one and two) as well as anatomical asplenia (case two). Only a single case reported here had received a prior pneumococcal vaccination. Of note, this patient (case two) had received vaccination with the 23-valent pneumococcal polysaccharide vaccine but was infected with serotype 23B, which is not covered by this vaccination.

## Conclusions

This case series highlights that S. pneumoniae is a rare but important cause of intra-abdominal infection. The disease is more common in females. Risk factors include an indwelling IUD, long-term steroid use or immunocompromise. Empiric therapy for a pneumococcal disease may include penicillin, amoxicillin, moxifloxacin or a third-generation cephalosporin. In patients presenting with clinical syndromes suggesting colitis, particularly if severe or affecting a high-risk group, ciprofloxacin or macrolides are commonly prescribed empirically. These empiric treatment regimens do not provide adequate coverage for S. pneumoniae, which is a rare pathogen in these settings. Nonetheless, it is important to consider this organism in the differential diagnosis particularly for patients with risk factors for an invasive pneumococcal infection or with a concomitant respiratory infection at presentation.
